# A Transformer-Based Spatiotemporal Fusion Network for Automatic Modulation Classification

**DOI:** 10.3390/e28090990

**Published:** 2026-09-04

**Authors:** Mingdong Xu, Guina Zhao, Yanrong Zhang, Dequan Zheng, Jinlong Liu

**Affiliations:** 1 School of Computer and Information Engineering, Harbin University of Commerce, Harbin 150028, China; xumingdong@hrbcu.edu.cn (M.X.); zhaoguinay25@s.hrbcu.edu.cn (G.Z.); 102699@hrbcu.edu.cn (Y.Z.); 2 Postdoctoral Research Station of Northeast Asia Service Outsourcing Research Center, Harbin University of Commerce, Harbin 150028, China; 3 Heilongjiang Provincial Key Laboratory of Fluid Engineering Equipment and Digital Intelligence Technology, Harbin University of Commerce, Harbin 150028, China; 4 School of Electronics and Information Engineering, Harbin Institute of Technology, Harbin 150001, China

**Keywords:** automatic modulation classification, spatiotemporal fusion, Transformer, deep learning, signal-to-noise ratio

## Abstract

Automatic modulation classification (AMC) suffers from performance degradation under low signal-to-noise ratio (SNR) conditions, where modulation characteristics are affected by noise and signals belonging to the same modulation family exhibit similar feature representations. To address these challenges, this paper proposes a Transformer-based spatiotemporal fusion network that jointly exploits local spatial waveform characteristics and temporal dependency information while leveraging the global context modeling capability of the Transformer to integrate complementary multi-dimensional features. The proposed architecture improves feature representation capability under different SNR conditions. Experimental results demonstrate that the proposed method achieves improved classification performance compared with comparative approaches under different SNR conditions. In particular, it achieves an overall classification accuracy of 82.45% over the SNR range from −10 to 18 dB, and an average accuracy of 95.55% at SNRs of 2 dB and above, showing improved classification performance in the low-to-medium SNR transition region.

## 1. Introduction

Automatic modulation classification (AMC) aims to identify the modulation format of a received radio signal without prior knowledge of the transmitter parameters [1]. It serves as an intermediate process between signal detection and demodulation and plays an important role in spectrum monitoring, cognitive radio, electronic reconnaissance, interference identification, and intelligent wireless receivers [2]. Reliable AMC allows a receiver to adapt its demodulation and decoding strategy according to the estimated signal type, thereby improving the autonomy and flexibility of communication systems [3].

Conventional automatic modulation classification methods are generally divided into likelihood-based and feature-based approaches [4]. Likelihood-based methods formulate modulation classification as a multiple-hypothesis testing problem and determine the modulation type by comparing the likelihood functions or likelihood ratios of the received signal under different modulation hypotheses [5,6]. Hameed et al. [7] investigated hybrid likelihood ratio test and quasi-hybrid likelihood ratio test methods when the signal amplitude, carrier phase, and noise power were unknown. Xu et al. [8] further reviewed and compared several likelihood-ratio-based classification strategies. These methods can provide reliable classification performance when the signal model and related parameters are sufficiently accurate, but they usually involve relatively high computational complexity and are sensitive to parameter estimation errors and model mismatch. Feature-based methods first extract characteristics such as instantaneous amplitude, phase, frequency, higher-order statistics, or cyclostationary features, and then use decision rules or machine learning classifiers to identify the modulation type [9,10]. Nandi and Azzouz [11] used manually designed statistical features to classify several analog and digital modulation signals. Swami and Sadler [12] applied higher-order cumulants to hierarchical digital modulation classification. Dobre et al. [13] and Orlic and Dukic [14] further investigated the use of cyclic cumulants and normalized higher-order cumulants for modulation classification. Although feature-based methods can reduce part of the computational burden, their performance depends on the design of handcrafted features [15]. Under low SNR, multipath fading, frequency offset, and other non-ideal conditions, the stability and discriminative capability of these features may decrease [16].

Deep learning can learn modulation-related feature representations directly from received signals, thereby reducing the dependence on manually designed features [17,18]. To address the difficulty of adapting handcrafted features to complex channel conditions, Jariwala and Captain [19] proposed a convolutional neural network (CNN)-based automatic modulation classification method that learns modulation features from received signals through convolutional operations. Zheng et al. [20] considered the limitation of fixed-length CNN inputs in processing variable-length signal sequences and proposed voting fusion, confidence fusion, and feature fusion methods to combine classification information from different signal segments. These studies show that CNNs can extract local waveform features from raw signals. However, because convolutional operations mainly process neighboring sampling positions through local receptive fields, their ability to model long-range temporal dependencies is limited [21,22]. Therefore, additional temporal modeling mechanisms are required to capture the sequential evolution characteristics of modulation signals. To further capture temporal relationships in signal sequences, Rajendran et al. [23] applied long short-term memory (LSTM) networks to wireless signal classification and used recurrent structures to learn dependencies among different sampling positions. Kumar et al. [24] proposed a residual CNN-LSTM model for modulation classification in orthogonal time-frequency space (OTFS) systems, where the CNN extracts local features and the LSTM models temporal variations in the signal sequence. Zhang et al. [25] developed a CNN-LSTM dual-stream architecture that separately processes in-phase/quadrature (I/Q) sequences and amplitude/phase representations and combines the two streams through feature interaction. Xu et al. [26] proposed a spatiotemporal multi-channel learning framework that uses one-dimensional CNNs, two-dimensional CNNs, and LSTMs to process individual and combined I/Q components and extract local and temporal features from different representations. Tahir et al. [27] proposed a hybrid fusion deep neural network (HFDNN), which integrates a Visual Geometry Group (VGG) network, LSTM-CNN, gated recurrent unit-convolutional neural network (GRU-CNN), and convolutional LSTM networks to perform modulation classification under additive noise, fading, carrier frequency offset, and phase noise conditions. To reduce the structural complexity and recurrent computation cost of LSTM networks, Liu et al. [28] proposed a modulation classification method that combines CNNs with an optimized gated recurrent unit (GRU). The CNN is used for feature extraction, while the GRU models temporal relationships in the signal sequence. Although the GRU reduces part of the structural complexity of the LSTM through a simplified gating mechanism, the recurrent branches in CNN-LSTM and CNN-GRU models still update their hidden states successively along the temporal dimension. Consequently, recurrent computations at different sampling positions cannot be fully parallelized, and information between distant samples must be propagated through multiple recurrent steps [29,30]. Moreover, although CNN-LSTM architectures can capture local and sequential patterns, they lack explicit global interaction modeling among all sampling positions. To model long-range relationships across the entire sampling sequence, Cai et al. [31] applied the Transformer to modulation classification and used self-attention to integrate global information from different sampling positions. Ma et al. [32] further proposed PCTNet, a parallel CNN-Transformer network in which the CNN branch extracts local waveform features and the Transformer branch models long-range dependencies, followed by the fusion of the outputs from the two branches. However, existing CNN-Transformer frameworks mainly focus on the combination of local waveform and global contextual information, while the temporal evolution patterns of modulation signals are not explicitly modeled. Although existing deep learning methods can learn discriminative information from different aspects of modulation signals, they still have limitations. Under complex channels and low SNR conditions, noise and channel distortions can reduce the reliability of representations obtained from a single feature domain, making modulation schemes with similar signal characteristics more difficult to distinguish [33,34].

To address the limitations of existing methods in jointly modeling local waveform characteristics, temporal evolution patterns, and global contextual relationships, this paper proposes a Transformer-based spatiotemporal fusion network, referred to as TSF-Net. The model first constructs a multi-dimensional representation from the original I/Q sequence and then extracts local waveform and temporal dependency features through two parallel feature-learning paths. The resulting features are aligned and fused in a common feature space. A global context encoder is then employed to model long-range interactions in the fused sequence. Finally, a sequence-level representation is used to classify eight digital modulation types.

The main contributions of this paper are summarized as follows:

1. A hierarchical spatiotemporal feature learning framework is developed for AMC, where CNN and LSTM branches are designed to capture local waveform characteristics and temporal evolution patterns from multi-dimensional signal representations constructed from the original I/Q sequences, respectively.
2. A Transformer-based global context refinement module is introduced after local and temporal feature fusion. It captures long-range dependencies among sampling positions and enhances the sequence-level representation by modeling global interactions within the fused feature space.
3. The proposed TSF-Net achieves improved modulation classification performance while maintaining a moderate parameter scale compared with existing hybrid architectures, demonstrating a favorable trade-off between classification accuracy and model complexity.

## 2. Materials and Methods

### 2.1. Proposed Transformer-Based Spatiotemporal Fusion Method

#### 2.1.1. Overall Architecture

The proposed TSF-Net is designed to learn complementary representations of modulation signals from a unified multi-dimensional signal representation. Specifically, the CNN branch captures local waveform variations among adjacent sampling positions, while the LSTM branch models temporal evolution patterns across the sampling sequence. The outputs of the two branches are then fused and further processed by a Transformer encoder to capture global contextual relationships among sampling positions.

**Figure 1:**
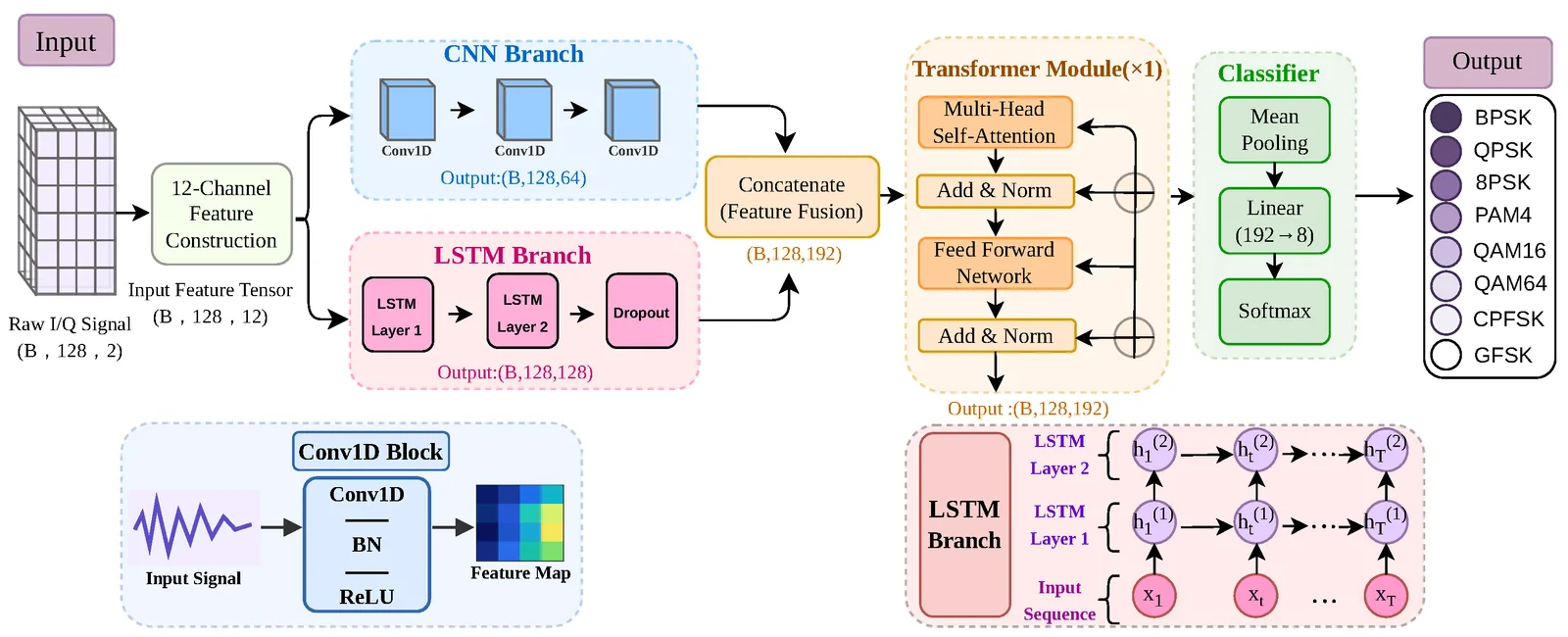
Overall architecture of the proposed Transformer-based spatiotemporal fusion network.

As shown in Figure 1, TSF-Net consists of three main feature-learning modules: a convolutional local waveform representation branch, an LSTM-based temporal dependency representation branch, and a Transformer-based global context modeling module. The original 128×2 I/Q sequence is first converted into a 128×12 feature tensor using the preprocessing method described in the previous subsection. The resulting feature tensor is simultaneously fed into the CNN and LSTM branches. The CNN branch extracts short-range waveform patterns, whereas the LSTM branch models the sequential dependencies among sampling positions. The two representations are concatenated along the feature dimension and subsequently processed by a Transformer encoder to capture long-range interactions. Finally, mean pooling and a fully connected layer are used to produce the classification scores of the eight modulation classes.

For a batch of preprocessed input samples, the model input is represented as

(1) $$X\in R^{B\times T\times C},$$

where *B* denotes the batch size, T=128 denotes the sequence length, and C=12 denotes the number of input feature channels. The twelve channels consist of the original I/Q components and the derived amplitude-, power-, and phase-related features. The following subsections describe the three feature-learning modules in detail.

#### 2.1.2. Local Waveform Representation Branch

The local waveform representation branch employs three one-dimensional convolutional layers to extract short-range modulation-related characteristics from the multi-dimensional signal representation. Since the convolutional kernels operate along the sampling dimension, this branch can capture local variations and correlations among adjacent sampling positions.

Let $F_{0}=X$. The convolutional operation of the *l*-th layer is expressed as

(2) $${\tilde{F}}_{l}=\mathrm{Conv1D}(F_{l-1};W_{l},b_{l}),$$

where $W_{l}$ and $b_{l}$ denote the convolutional kernel and bias of the *l*-th layer, respectively. Group normalization is applied after each convolutional operation to stabilize feature distributions during training. For the first two convolutional layers, the normalized outputs are further processed by the rectified linear unit (ReLU) activation function. The normalized output of the third convolutional layer is retained as the final local feature representation, denoted as $F_{\mathrm{loc}}$. The three convolutional layers use 16, 32, and 64 output channels, respectively. Each layer employs a kernel size of 3, a stride of 1, and a padding size of 1. Therefore, the temporal length remains unchanged throughout the branch. The final local feature representation is

(3) $$F_{\mathrm{loc}}\in R^{B\times 128\times 64}.$$

The detailed configuration of the local waveform representation branch is summarized in Table 1.

#### 2.1.3. Temporal Dependency Representation Branch

The temporal dependency representation branch consists of two stacked unidirectional LSTM layers. It is designed to model the sequential evolution of modulation-related features over the 128 sampling positions. Unlike the CNN branch, which operates on local neighborhoods, the LSTM branch maintains recurrent hidden states to capture dependencies among different sampling positions.

The input sequence is represented as

(4) $$X=\left[x_{1},x_{2},\ldots ,x_{T}\right],\;x_{t}\in R^{12}.$$

where $x_{t}$ denotes the twelve-dimensional feature vector at the *t*-th sampling position. The LSTM layers follow the standard recurrent formulation to update hidden states and cell states, enabling the modeling of temporal dependencies among sampling positions.

Both LSTM layers use a hidden dimension of 128. Instead of using only the final hidden state, the complete hidden-state sequence generated by the second LSTM layer is retained as the temporal representation

(5) $$F_{\mathrm{tem}}=\left[h_{1}^{\left(2\right)},h_{2}^{\left(2\right)},\ldots ,h_{T}^{\left(2\right)}\right]\in R^{B\times 128\times 128}.$$

A dropout layer with a rate of 0.1 is applied to the output sequence to reduce overfitting. The detailed configuration is listed in Table 2.

#### 2.1.4. Spatiotemporal Feature Fusion and Global Context Modeling

The local and temporal representations extracted from the CNN and LSTM branches are first fused along the feature dimension. Since the CNN and LSTM branches produce 64- and 128-dimensional feature representations, respectively, the fused feature dimension is 192. The fusion operation is formulated as

(6) $$F_{\mathrm{fus}}=\mathrm{Concat}(F_{\mathrm{loc}},F_{\mathrm{tem}})\in R^{B\times 128\times 192}.$$

The fused sequence is then input into a one-layer Transformer encoder to capture long-range dependencies among different sampling positions. Unlike convolutional operations that mainly focus on local neighborhoods and recurrent structures that update information sequentially, the self-attention mechanism in the Transformer encoder establishes interactions among all sampling positions simultaneously. The self-attention operation follows the standard Transformer formulation, and four attention heads are employed in this study.

After the multi-head self-attention module, a residual connection and layer normalization are applied to enhance feature propagation and stabilize the training process. The output of the attention sub-layer can be expressed as

(7) $$H=\mathrm{LayerNorm}[F_{\mathrm{fus}}+\mathrm{Dropout}(\mathrm{MHA}(F_{\mathrm{fus}}))].$$

The position-wise feed-forward network (FFN) consists of two fully connected layers with an intermediate feature dimension of 2048. Instead of explicitly rewriting the standard feed-forward formulation, the Transformer encoder follows the original Transformer architecture. The final global representation is obtained by applying another residual connection and layer normalization

(8) $$F_{\mathrm{glo}}=\mathrm{LayerNorm}[H+\mathrm{Dropout}(\mathrm{FFN}(H))].$$

where the final output feature representation is

(9) $$F_{\mathrm{glo}}\in R^{B\times 128\times 192}.$$

No additional positional encoding is introduced in this network. The sequential ordering information has already been embedded into the fused representation through the convolutional and recurrent branches before applying global self-attention. The detailed configuration of the feature fusion, Transformer encoder, and classification modules is summarized in Table 3.

#### 2.1.5. Classification Layer

The Transformer output is converted into a fixed-length representation by applying mean pooling along the sequence dimension

(10) $$z=\frac{1}{T}\sum_{t=1}^{T}F_{\mathrm{glo}}\left(:,t,:\right),\;z\in R^{B\times 192}.$$

The pooled feature is then fed into a fully connected layer to generate classification logits corresponding to the eight modulation classes. The Softmax function is applied to convert the logits into class probabilities during inference. The modulation class with the highest predicted probability is selected as the final classification result.

### 2.2. Dataset and Preprocessing

This study uses the RadioML2016.10a dataset [35], which contains complex baseband signals generated under different modulation and SNR conditions. Each signal sample consists of 128 sampling positions, with each position represented by an in-phase component and a quadrature component. The original data are therefore arranged as an I/Q sequence

(11) $$X_{\mathrm{IQ}}=\left[\begin{array}{cc} I_{1} & Q_{1}\\ I_{2} & Q_{2}\\ \vdots & \vdots \\ I_{128} & Q_{128} \end{array}\right]\in R^{128\times 2}.$$

For the AMC task considered in this study, eight digital modulation schemes are selected from the original dataset, namely binary phase-shift keying (BPSK), quadrature phase-shift keying (QPSK), 8-ary phase-shift keying (8PSK), four-level pulse amplitude modulation (PAM4), 16-ary quadrature amplitude modulation (QAM16), 64-ary quadrature amplitude modulation (QAM64), continuous-phase frequency-shift keying (CPFSK), and Gaussian frequency-shift keying (GFSK). Only samples with SNR values ranging from −10 to 18 dB at intervals of 2 dB are retained. Each modulation–SNR combination contains 1000 samples, resulting in a total of 120,000 samples. The selected samples are divided into training, validation, and testing sets using a ratio of 3:1:1. Stratified splitting is performed according to the combination of modulation label and SNR, ensuring that every subset maintains consistent modulation-class and SNR distributions. Consequently, the training, validation, and testing sets contain 72,000, 24,000, and 24,000 samples, respectively.

To provide the network with complementary representations of the modulation signals, each original I/Q sequence is expanded into a 12-channel feature tensor. For the *t*th sampling position, the amplitude, power, and phase are calculated as

(12) $$A_{t}=\sqrt{I_{t}^{2}+Q_{t}^{2}+\varepsilon },\;P_{t}=I_{t}^{2}+Q_{t}^{2},\;\phi_{t}=\mathrm{atan}2\left(Q_{t},I_{t}\right),$$

where ε is a small constant used to maintain numerical stability. The normalized amplitude and power are defined as

(13) $${\hat{A}}_{t}=\frac{A_{t}}{\frac{1}{T}\sum_{\tau =1}^{T}A_{\tau }+\varepsilon },\;{\hat{P}}_{t}=\frac{P_{t}}{\frac{1}{T}\sum_{\tau =1}^{T}P_{\tau }+\varepsilon },$$

where T=128. After phase unwrapping, the phase difference between adjacent sampling positions is computed and wrapped into the interval [−π,π) to remove phase ambiguity

(14) $$\Delta \phi_{t}=\left((\phi_{t}^{u}-\phi_{t-1}^{u}+\pi )\;\mathrm{mod}\;2\pi \right)-\pi$$

where $\phi_{t}^{u}$ denotes the unwrapped phase, and the modulo operation ensures that the phase difference is represented within the principal phase interval.

(15) $$f_{t}=\left[I_{t},\;Q_{t},\;A_{t},\;\log\left(1+A_{t}\right),\;P_{t},\;{\hat{A}}_{t},\;{\hat{P}}_{t},\;\sin\phi_{t},\;\cos\phi_{t},\;\Delta \phi_{t},\;\sin\Delta \phi_{t},\;\cos\Delta \phi_{t}\right].$$

Accordingly, each signal sample is transformed from an original 128×2 I/Q sequence into a 128×12 feature tensor. The original I/Q components and temporal sampling order are retained, while the additional channels describe the amplitude, power, phase, and phase-transition characteristics of the signal.

Finally, channel-wise standardization is performed using statistics calculated exclusively from the training set. For each feature channel, the training-set mean and standard deviation are calculated over all training samples and sampling positions. Each feature value is then standardized according to

(16) $${\tilde{F}}_{n,t,c}=\frac{F_{n,t,c}-\mu_{c}}{\sigma_{c}+\varepsilon }$$

where $\mu_{c}$ and $\sigma_{c}$ denote the training-set mean and standard deviation of the *c*th feature channel, respectively. The same training-set statistics are applied to the validation and testing sets to avoid information leakage. This preprocessing procedure provides the model with complementary waveform and phase-related information while maintaining consistent feature scales across the three subsets. The dataset configuration used in this study is summarized in Table 4.

### 2.3. Model Training

The proposed network is trained using the multiclass cross-entropy loss. Let *L* denote the number of samples in a mini-batch, $y_{l,m}$ denote the label indicator of the *l*-th sample for the *m*-th modulation class, and $p_{l,m}$ denote the corresponding predicted probability. The classification loss is defined as

(17) $$L_{\mathrm{ce}}=-\frac{1}{L}\sum_{l=1}^{L}\sum_{m=1}^{8}y_{l,m}\log p_{l,m}.$$

The trainable parameters of TSF-Net, denoted by θ, are optimized by minimizing the classification loss

(18) $$\theta^{*}=\arg\min_{\theta }L_{\mathrm{ce}}\left(\theta \right).$$

The model parameters are updated through backpropagation using the AdamW optimizer. The initial learning rate is set to $2\times 10^{-4}$, and a weight decay of $2\times 10^{-4}$ is applied during optimization. To improve the stability of the joint training process, gradient clipping is employed, with the maximum gradient norm set to 1.0.

A dynamic learning-rate adjustment strategy based on ReduceLROnPlateau is adopted. The validation loss is evaluated after each epoch. When the validation loss does not improve for four consecutive epochs, the learning rate is reduced by a factor of 0.5, with the minimum learning rate set to $1\times 10^{-6}$.

The model performance is evaluated on the validation set after each training epoch, and the model checkpoint with the best validation performance is retained. Early stopping is applied when the validation performance does not improve for 12 consecutive epochs. The retained model is finally evaluated on the independent testing set.

## 3. Simulation Results

### 3.1. Experimental Setup and Evaluation Metrics

The proposed TSF-Net is comprehensively evaluated using overall accuracy, macro-F1, weighted-F1, per-class accuracy, and row-normalized confusion matrices. In addition, its classification accuracy is compared with DCN-based AMC [36], DNN-AMC2 [37], GCN Feature Fusion [38], and HFDNN [27] over the same SNR range. The experiments are conducted from three main perspectives. First, the variation in classification performance under different SNR conditions is investigated to evaluate the robustness of the proposed model to noise. Second, per-class accuracy and confusion matrices are analyzed to examine the classification performance of different modulation types and identify modulation classes that remain difficult to distinguish. Finally, comparisons with existing methods are performed to assess the performance advantages of the proposed spatiotemporal fusion representation for automatic modulation classification.

### 3.2. Classification Accuracy Versus SNR

**Figure 2:**
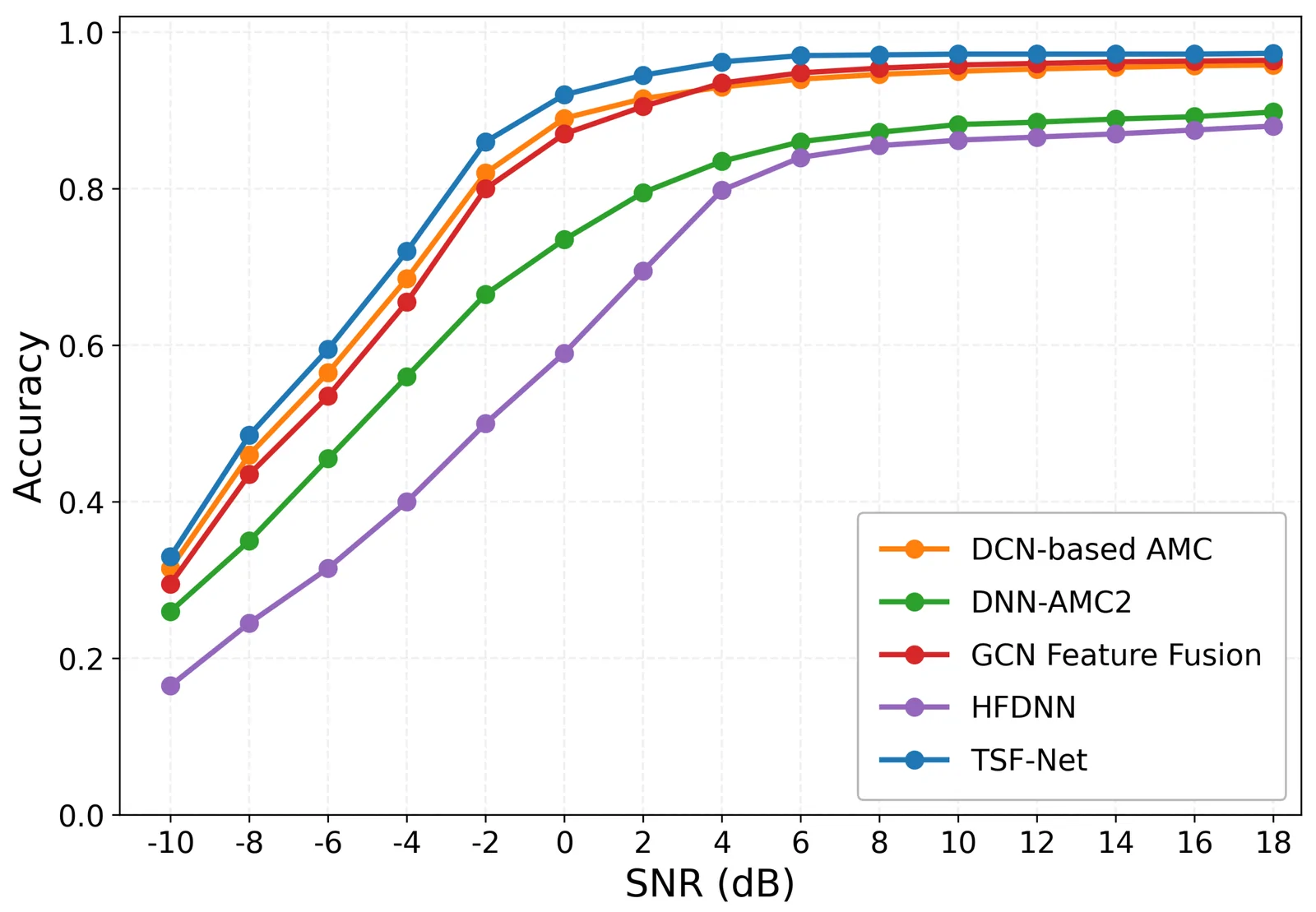
Classification accuracy of the proposed method and comparison methods at different SNR values.

Figure 2 compares the classification accuracy of TSF-Net and the comparison methods under different SNR conditions, aiming to evaluate their performance variations with signal quality. As shown in Figure 2, the classification accuracy of all methods generally increases with the SNR, while TSF-Net maintains the highest accuracy over the evaluated SNR range. In the low-SNR region from −10 to −4 dB, the performance differences among the leading methods are relatively small, but TSF-Net consistently remains ahead of DCN-based AMC and GCN Feature Fusion. At −10 dB, TSF-Net achieves an accuracy of approximately 33%, compared with about 31.5% for DCN-based AMC and 29.5% for GCN Feature Fusion. As the SNR increases, the accuracy of TSF-Net rises steadily to approximately 48.5%, 59.5%, and 72% at −8, −6, and −4 dB, respectively. At −2 dB, its accuracy reaches approximately 86%, exceeding DCN-based AMC, GCN Feature Fusion, DNN-AMC2, and HFDNN by about 4, 6, 19, and 36 percentage points, respectively. The accuracy further increases to approximately 92%, 94.5%, and 96.2% at 0, 2, and 4 dB, indicating that TSF-Net maintains a consistent advantage as the modulation characteristics become more distinguishable with increasing SNR.

From approximately 6 dB onward, the accuracy of TSF-Net stabilizes at around 97%, showing that the model reaches a relatively stable performance level earlier than the comparison methods. GCN Feature Fusion becomes the closest competing method in the medium- and high-SNR regions, followed by DCN-based AMC, while DNN-AMC2 and HFDNN exhibit lower saturation levels. At 18 dB, TSF-Net achieves an accuracy of approximately 97.3%, which is about 0.9, 1.5, 7.5, and 9.3 percentage points higher than those of GCN Feature Fusion, DCN-based AMC, DNN-AMC2, and HFDNN, respectively. Although the performance gap between TSF-Net and the strongest comparison methods becomes smaller at high SNR, TSF-Net maintains the highest classification accuracy throughout the evaluated range. Overall, these results indicate that the proposed spatiotemporal fusion architecture provides consistent classification performance under different noise conditions, with its advantage being particularly evident in the low-to-medium-SNR transition region while maintaining a high and stable accuracy level under medium- and high-SNR conditions.

### 3.3. Overall Performance Metrics

**Figure 3:**
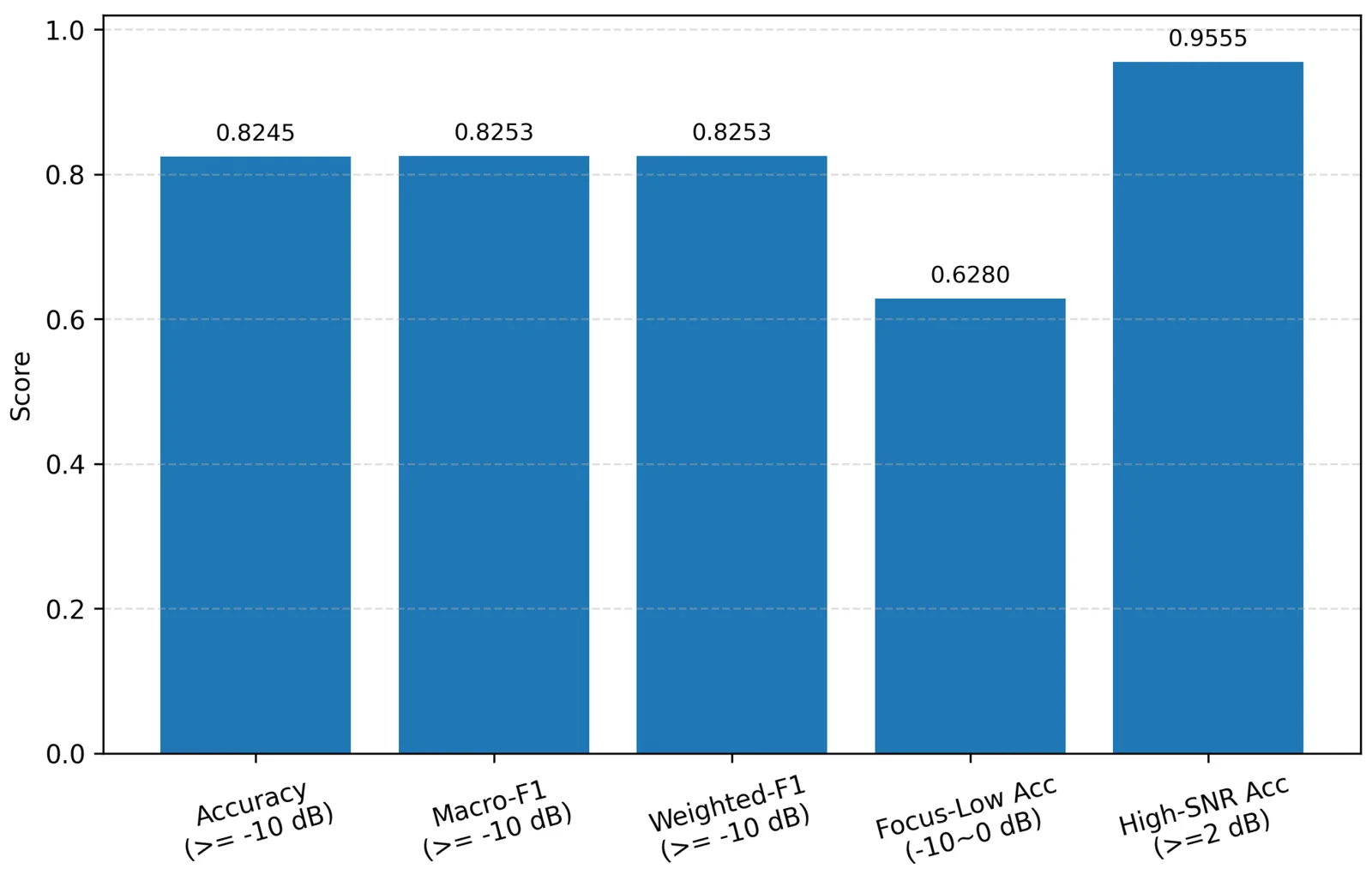
Overall accuracy, macro-F1, and weighted-F1 scores of TSF-Net.

As shown in Figure 3, TSF-Net achieves an overall accuracy of 82.45%, a macro-F1 score of 82.53%, and a weighted-F1 score of 82.53% over the SNR range from −10 to 18 dB. The small difference among these three metrics indicates that the overall evaluation is not dominated by a limited number of modulation classes. In particular, the nearly identical macro-F1 and weighted-F1 scores are consistent with the balanced class distribution of the test set. Nevertheless, the subsequent class-wise results show that the classification performance still varies among different modulation types, indicating that similar values of the overall evaluation metrics do not imply identical classification performance across all classes.

A clear performance difference can also be observed between the low- and medium-to-high-SNR regions. In the SNR range from −10 to 0 dB, the average classification accuracy is 62.80%, whereas it increases to 95.55% when the SNR is 2 dB or higher, corresponding to an improvement of 32.75 percentage points. This difference indicates that severe noise remains an important factor limiting the overall classification performance, as it reduces the discriminability of waveform and temporal characteristics among different modulation types. By contrast, when the SNR reaches 2 dB or higher, the average classification accuracy exceeds 95%, indicating that the modulation-related features become more distinguishable as the influence of noise decreases. At −10 dB, TSF-Net achieves an accuracy of approximately 33%, which remains higher than those of the considered comparison methods under the same condition. This result indicates that extremely low-SNR modulation classification remains a challenging task because strong noise weakens the discriminability of waveform, amplitude, and phase characteristics. The influence is more evident for modulation formats with similar signal structures, such as QPSK/8PSK and QAM16/QAM64. Nevertheless, as the SNR increases from −10 dB to the low-to-medium SNR region, the classification accuracy of TSF-Net improves rapidly and maintains an advantage over the comparison methods. These observations suggest that the proposed spatiotemporal representation can improve feature discrimination under noisy conditions, while further enhancement is still desirable for extremely low SNR scenarios. Overall, these results show that TSF-Net maintains stable overall classification performance across the evaluated SNR range and achieves high classification accuracy under medium- and high-SNR conditions, while further improvement is still required under low SNR conditions.

### 3.4. Per-Class Accuracy Versus SNR

**Figure 4:**
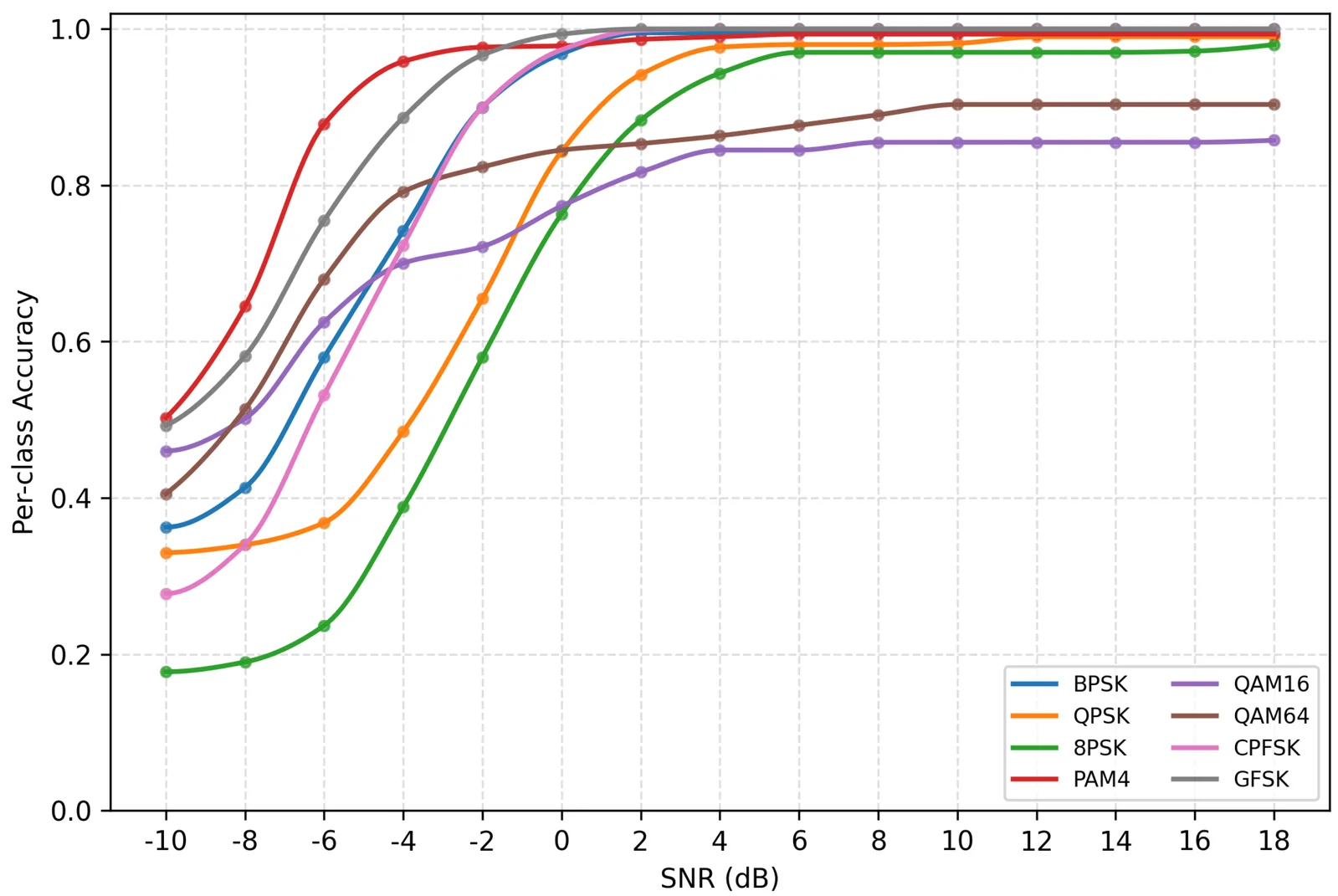
Per-class classification accuracy at different SNR values.

Figure 4 presents the per-class classification accuracy of the eight modulation types under different SNR conditions, allowing the classification characteristics of individual modulation schemes to be examined. As shown in Figure 4, the classification accuracy of all modulation classes increases with the SNR, although the improvement rates differ across modulation types. At −10 dB, PAM4 and GFSK achieve the highest accuracies of approximately 50%, followed by QAM16 and QAM64 at approximately 46% and 40%, respectively, whereas 8PSK exhibits the lowest accuracy of approximately 18%. PAM4 shows the fastest improvement in the low-SNR region, increasing from approximately 50% at −10 dB to more than 95% at −4 dB. GFSK also improves rapidly and reaches approximately 97% at −2 dB. Similarly, the accuracies of BPSK and CPFSK increase from approximately 36% and 28% at −10 dB to approximately 96–97% at 0 dB, and both approach 100% when the SNR reaches 2–4 dB. These results indicate that the proposed feature-fusion architecture can effectively recover discriminative waveform and temporal characteristics as the influence of noise decreases.

QPSK and 8PSK exhibit slower performance improvement because their discriminative phase characteristics are more severely affected under low SNR conditions. At 0 dB, their accuracies are approximately 85% and 77%, respectively, but they increase to approximately 98.5% and 97% at 6 dB. In contrast, QAM16 and QAM64 reach lower saturation levels than the other modulation classes. Their high-SNR accuracies stabilize at approximately 85.5% and 90.5%, respectively, suggesting that residual ambiguity remains between modulation patterns with similar amplitude–phase structures even when the noise level is reduced. Nevertheless, from approximately 6 dB onward, six of the eight modulation classes achieve accuracies of about 97% or higher, and the performance curves remain stable as the SNR further increases. Overall, the results demonstrate that TSF-Net can effectively integrate local waveform features, temporal dependencies, and global contextual information for different modulation types, while the classification of higher-order QAM signals and modulation signals under low SNR conditions remains the main direction for further improvement.

### 3.5. Confusion Matrix Analysis

**Figure 5:**
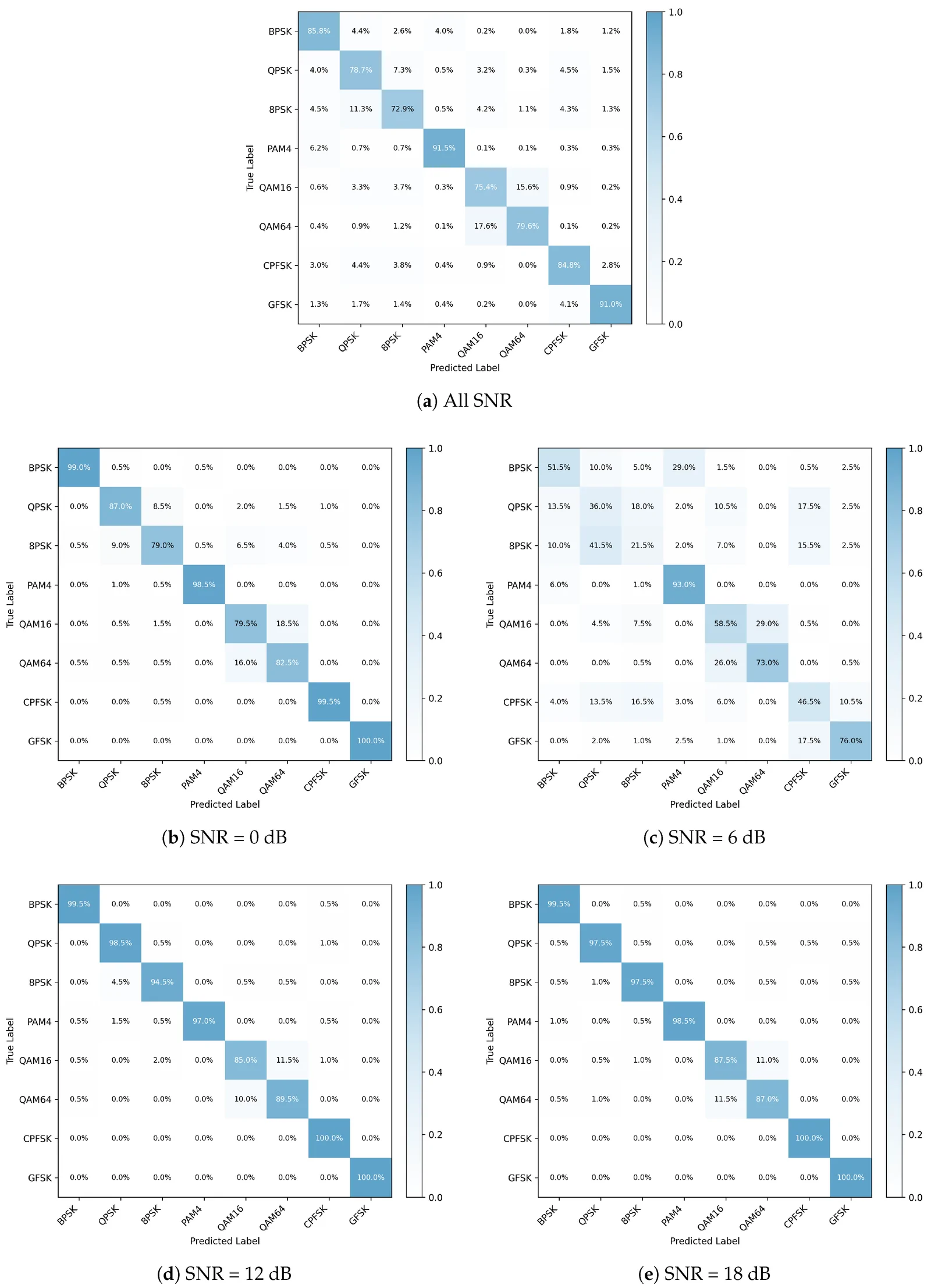
Row-normalized confusion matrices of TSF-Net under different SNR conditions: (**a**) all evaluated SNR values; (**b**) 0 dB; (**c**) 6 dB; (**d**) 12 dB; (**e**) 18 dB.

As shown in Figure 5, the predictions of TSF-Net become progressively concentrated along the main diagonal as the SNR increases. Since the confusion matrices are row-normalized, each diagonal entry represents the recall of the corresponding modulation class. For the results aggregated over all evaluated SNR values in Figure 5a, the diagonal values range from 72.9% to 91.5%. PAM4 and GFSK achieve the highest recalls of 91.5% and 91.0%, respectively, whereas 8PSK and QAM16 show lower recalls of 72.9% and 75.4%. The classification errors are mainly concentrated among modulation formats with similar signal characteristics. In particular, 11.3% of 8PSK samples are misclassified as QPSK, while 7.3% of QPSK samples are classified as 8PSK. A more pronounced bidirectional confusion is observed between QAM16 and QAM64, with misclassification rates of 15.6% and 17.6%, respectively. By comparison, the mutual confusion between CPFSK and GFSK is limited to 2.8% and 4.1%. These results indicate that the classification errors are not uniformly distributed across the eight modulation types but are mainly associated with neighboring PSK orders and QAM formats with similar amplitude–phase characteristics.

The SNR-specific confusion matrices further illustrate how these classification errors evolve as the signal quality improves. At 0 dB, BPSK, PAM4, CPFSK, and GFSK already achieve recalls of 98.5–100%. However, QPSK and 8PSK still exhibit mutual confusion rates of 8.5% and 9.0%, respectively, while QAM16 and QAM64 are confused with each other at rates of 18.5% and 16.0%. When the SNR increases to 6 dB, six of the eight modulation classes achieve recalls of at least 97%. The mutual confusion between QPSK and 8PSK decreases to 1.5% and 2.0%, indicating that their phase-related differences become more distinguishable as the influence of noise decreases. This classification pattern is generally maintained at 12 and 18 dB. Nevertheless, QAM16 and QAM64 remain the primary sources of residual classification errors: their recalls remain within approximately 85–89.5%, and their mutual misclassification rates remain around 10–12% even under high-SNR conditions. Overall, the confusion-matrix results show that increasing the SNR substantially reduces the ambiguity between most modulation classes, particularly QPSK and 8PSK, whereas the distinction between QAM16 and QAM64 remains comparatively difficult because of their similar amplitude–phase characteristics.

### 3.6. Model Complexity Analysis

Table 5 compares the parameter counts of the evaluated AMC models. DNN-AMC2 and GCN Feature Fusion contain 0.116 M and 0.048 M parameters, respectively, and therefore have relatively small model sizes. DCN-based AMC has the largest parameter count of 4.713 M, while HFDNN contains 1.179 M parameters. In comparison, the proposed TSF-Net contains 1.146 M parameters, which is approximately 75.7% fewer than DCN-based AMC and slightly fewer than HFDNN. Although the parameter count of TSF-Net is higher than those of DNN-AMC2 and GCN Feature Fusion, it remains within a moderate range.

More importantly, the preceding comparison shows that TSF-Net maintains the highest classification accuracy over the evaluated SNR range. Its performance advantage becomes more evident in the transition from low to medium SNR, while a high and stable accuracy level is maintained under medium- and high SNR conditions. Compared with DCN-based AMC, TSF-Net achieves higher classification accuracy with a substantially smaller parameter count, while its parameter scale remains comparable to that of HFDNN. Although DNN-AMC2 and GCN Feature Fusion require fewer parameters, their classification accuracies are lower than that of TSF-Net over the evaluated SNR range.

Overall, these results indicate that TSF-Net achieves a favorable trade-off between classification performance and model complexity. Although its parameter count is higher than those of DNN-AMC2 and GCN Feature Fusion, it remains lower than DCN-based AMC and comparable to HFDNN while achieving higher classification accuracy over the evaluated SNR range.

### 3.7. Ablation Study

To evaluate the contribution of each core component and the effectiveness of the parallel feature extraction strategy in TSF-Net, ablation experiments were conducted using three reduced architectures: parallel CNN–LSTM, serial CNN–Transformer, and serial LSTM–Transformer. As shown in Table 6, the complete TSF-Net achieves the best performance, with an overall accuracy of 82.45% over the SNR range from −10 to 18 dB and an average accuracy of 95.55% at SNRs of 2 dB and above. When the Transformer module is removed, the parallel CNN–LSTM model obtains an overall accuracy of 80.26%, indicating that global contextual modeling provides complementary information beyond local waveform and temporal representations. In comparison, the serial CNN–Transformer architecture achieves a lower accuracy of 79.43%. In this serial configuration, the input sequence is first transformed by the CNN before being processed by the Transformer, which may weaken part of the fine-grained temporal information during the preceding local feature transformation. By contrast, the parallel CNN and LSTM branches in TSF-Net independently preserve local waveform characteristics and temporal evolution patterns before feature fusion, thereby providing more complementary information for subsequent global dependency modeling. The serial LSTM–Transformer model exhibits the lowest accuracy of 78.54%. Both LSTM and Transformer primarily focus on modeling sequential dependencies at different ranges, with the LSTM capturing relatively local and recurrent temporal relationships and the Transformer modeling long-range dependencies. Without the CNN branch, however, the network lacks a dedicated mechanism for extracting local waveform and spatial feature patterns from the multi-channel signal representation, resulting in reduced feature complementarity and the largest performance degradation among the three ablation variants.

It can also be observed that the performance differences among the different architectures become smaller at SNRs of 2 dB and above. Specifically, the average accuracies of the parallel CNN–LSTM, serial CNN–Transformer, serial LSTM–Transformer, and complete TSF-Net are 94.68%, 93.84%, 93.22%, and 95.55%, respectively. As the SNR increases, modulation-related characteristics become more distinguishable and are less severely disturbed by noise, allowing even reduced network configurations to extract sufficient discriminative information. In contrast, under low-to-medium-SNR conditions, useful waveform and temporal characteristics are more easily obscured by noise, and the complementary extraction of local, temporal, and global information becomes increasingly important. Therefore, the larger performance advantage of the complete TSF-Net over the reduced variants across the full SNR range demonstrates the effectiveness of combining parallel CNN–LSTM feature extraction with subsequent Transformer-based global dependency modeling, particularly under challenging noise conditions.

### 3.8. Generalization Study

To further examine the applicability of TSF-Net under a different dataset configuration, additional experiments were conducted on an 11-class subset of RadioML2018.01A, including OOK, 4ASK, BPSK, QPSK, 8PSK, 16QAM, AM-SSB-SC, AM-DSB-SC, FM, GMSK, and OQPSK [29]. Compared with RadioML2016.10a, each sample in RadioML2018.01A contains a longer I/Q sequence of 1024 sampling points. The SNR range was set from −10 to 18 dB with an interval of 2 dB, and the samples were divided into training, validation, and testing sets with a ratio of 60:20:20. The same 12-channel signal representation and the main TSF-Net architecture were retained to examine the model under different modulation classes and input sequence lengths.

**Figure 6:**
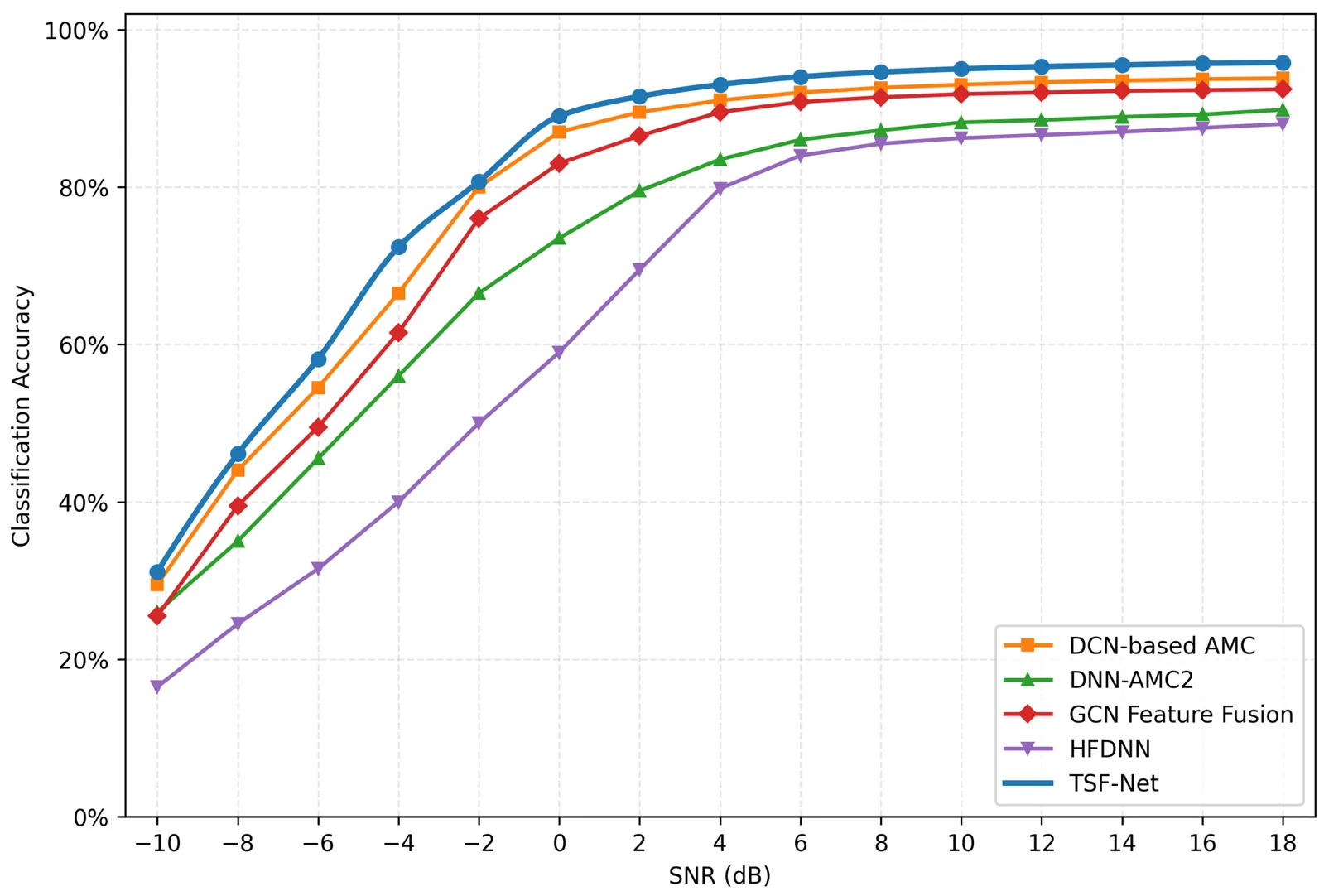
Classification accuracy of TSF-Net and comparison methods on the RadioML2018.01A 11-class subset under different SNR conditions.

Figure 6 compares the classification accuracy of TSF-Net with DCN-based AMC, DNN-AMC2, GCN Feature Fusion, and HFDNN on the RadioML2018.01A subset under different SNR conditions. As shown in Figure 6, the classification accuracy of all methods increases with SNR, while TSF-Net maintains the highest accuracy over the evaluated SNR range. Its accuracy increases from approximately 31% at −10 dB to about 58% at −6 dB and 81% at −2 dB, and further reaches approximately 89% at 0 dB. When the SNR is 2 dB or higher, the accuracy exceeds 90% and gradually approaches a stable level. DCN-based AMC and GCN Feature Fusion show relatively close performance to TSF-Net in the medium- and high-SNR regions, whereas DNN-AMC2 and HFDNN remain at lower accuracy levels. These results show that TSF-Net maintains a similar accuracy-versus-SNR trend on RadioML2018.01A and can be applied to a dataset with different modulation classes and a longer input sequence.

## 4. Conclusions

In this study, a Transformer-based spatiotemporal fusion network, termed TSF-Net, is proposed for automatic modulation classification by jointly modeling local waveform characteristics, temporal dependencies, and global contextual information. The proposed framework combines parallel CNN and LSTM branches with a subsequent Transformer encoder, enabling complementary feature extraction from multiple perspectives and improving the representation of modulation-related characteristics under varying noise conditions. Experimental results over the SNR range from −10 to 18 dB demonstrate that TSF-Net achieves an overall classification accuracy of 82.45%, together with a Macro-F1 score of 82.53% and a Weighted-F1 score of 82.53%. Among the considered comparison methods, TSF-Net consistently achieves the highest classification accuracy across the evaluated SNR range. Its performance improves rapidly as the SNR increases, rising from approximately 33% at −10 dB to 86% at −2 dB, exceeding 96% at 4 dB, and remaining stable at around 97% from approximately 6 dB onward. When the SNR is no lower than 2 dB, the average classification accuracy reaches 95.55%.

The ablation study further verifies the effectiveness of the proposed architecture. The complete TSF-Net outperforms the reduced CNN–LSTM, CNN–Transformer, and LSTM–Transformer variants, demonstrating that the three components provide complementary local, temporal, and global representations rather than redundant feature transformations. In particular, the parallel CNN–LSTM structure preserves local waveform and temporal evolution information independently before feature fusion, while the subsequent Transformer module further captures long-range contextual dependencies. The relatively smaller performance differences among the ablation variants at higher SNRs also suggest that the advantage of multi-level feature fusion is more pronounced under challenging noise conditions. In terms of model complexity, TSF-Net contains approximately 1.146 M parameters, which is about 75.7% fewer than DCN-based AMC and slightly fewer than HFDNN. Although its parameter count is higher than those of DNN-AMC2 and GCN Feature Fusion, TSF-Net maintains a moderate model size while achieving higher classification accuracy over the evaluated SNR range. Overall, these results indicate that TSF-Net provides a favorable balance between recognition performance, robustness, and model complexity.

Despite these improvements, modulation classification under extremely low-SNR conditions remains challenging because strong noise can substantially weaken the discriminability of waveform, amplitude, phase, and temporal characteristics. In addition, residual confusion remains between modulation formats with similar signal structures. Future work will therefore focus on improving feature robustness in extremely noisy environments, enhancing the discrimination of highly similar modulation classes, and further reducing computational and parameter complexity for deployment in resource-constrained intelligent communication systems.

## Figures and Tables

**Table 1:** Configuration of the local waveform representation branch.

Layer	Channel	Kernel/Stride/Pad.	Groups	Activation
Conv1D-1	12→16	3/1/1	4	ReLU
Conv1D-2	16→32	3/1/1	8	ReLU
Conv1D-3	32→64	3/1/1	8	–

**Table 2:** Configuration of the temporal dependency representation branch.

Layer	Input	Hidden Dim.	Direction	Layers
LSTM-1	12	128	Uni.	1
LSTM-2	128	128	Uni.	1
Dropout	128	128	–	–

**Table 3:** Configuration of the feature-fusion, Transformer, and classification modules.

Module	Input Dimension	Output Dimension	Main Configuration	Activation
Concatenation	64+128	192	Feature-axis concatenation	–
Multi-head self-attention	192	192	4 heads, $d_{k}=48$	Softmax
Feed-forward network	192	192	192→2048→192	ReLU
Layer normalization	192	192	Two normalization layers	–
Mean pooling	192	192	Average over 128 sampling positions	–
Linear classifier	192	8	192→8	–
Softmax	8	8	Class-probability normalization	Softmax

**Table 4:** Dataset configuration used in the experiments.

Item	Configuration
Dataset	RadioML2016.10a
Modulation classes	8
SNR range	−10 to 18 dB
SNR interval	2 dB
Samples per class–SNR pair	1000
Total number of samples	120,000
Training/validation/testing ratio	3:1:1
Training samples	72,000
Validation samples	24,000
Testing samples	24,000
Original sample dimension	128×2
Model input dimension	128×12

**Table 5:** Model parameter comparison.

Model	Parameters (M)
DNN-AMC2	0.116
GCN Feature Fusion	0.048
DCN-based AMC	4.713
HFDNN	1.179
TSF-Net	1.146

**Table 6:** Ablation study of different components in TSF-Net.

Model	CNN	LSTM	Transformer	Accuracy (%)	Accuracy (%)
				(−10–18 dB)	(SNR ≥2 dB)
Parallel CNN–LSTM	✓	✓	–	80.26	94.68
Serial CNN–Transformer	✓	–	✓	79.43	93.84
Serial LSTM–Transformer	–	✓	✓	78.54	93.22
TSF-Net	✓	✓	✓	82.45	95.55

## Data Availability

The data presented in this paper are available after contacting the corresponding author.

## Abbreviations

The following abbreviations are used in this manuscript:

AMC	Automatic modulation classification
SNR	Signal-to-noise ratio
I/Q	In-phase/quadrature
CNN	Convolutional neural network
LSTM	Long short-term memory
MHSA	Multi-head self-attention
FFN	Feed-forward network
PSK	Phase-shift keying
QAM	Quadrature amplitude modulation
FSK	Frequency-shift keying
